# Coalition Policy-Making under Constraints: Examining the Role of Preferences and Institutions

**DOI:** 10.1080/01402382.2013.841069

**Published:** 2013-10-09

**Authors:** Katrin Schermann, Laurenz Ennser-Jedenastik

## Abstract

*While much has been written about the formation and termination of coalitions, comparatively little attention has been paid to the policy output of multiparty governments. The present study attempts to narrow this research gap by analysing policy-making in three Austrian coalition governments between 1999 and 2008. Drawing on the party mandate literature, a manually coded textual analysis of election manifestos is conducted that yields a dataset containing over 1,100 pledges. The fulfilment of these pledges is taken as the dependent variable in a multivariate analysis. The results indicate that institutional determinants (adoption in the coalition agreement, ministerial control, and policy status quo) significantly influence the chances of pledge fulfilment and thus present a powerful predictor of coalition policy output. By contrast, factors related to parties’ preferences (consensus between parties, policy distance, pledge saliency, and majority support in parliament) do not have an impact.*

In parliamentary democracies with proportional electoral systems, the necessity to form coalition governments regularly subjects the translation of party platforms into government policy to the uncertainties of inter-party bargaining. This raises the question as to how parties in coalition governments arrive at a common policy agenda in the face of potentially divergent preferences.

Curiously, the policy output of multiparty governments has not been examined even nearly as extensively as their formation (Axelrod 1970; de Swaan 1973; Martin and Stevenson 2001; Riker 1962; Sened 1996), the allocation of government portfolios (Browne and Feste 1975; Browne and Franklin 1973; Browne and Frendreis 1980; Warwick and Druckman 2001, 2006), and their termination (Diermeier and Merlo 2000; Diermeier and Stevenson 1999; Laver 2003; Warwick 1994).

The most notable exceptions are extant studies of legislative output (Bräuninger and Debus 2009; Martin 2004; Martin and Vanberg 2004, 2005, 2011) that have stayed closer to the theoretical framework provided by coalition theory, and some studies on pledge fulfilment in multiparty governments that have come out of the party mandate literature (Costello and Thomson 2008; Kostadinova 2013; Mansergh and Thomson 2007; Moury 2011b; Thomson 2001).

We add to this research on coalition policy-making by examining the fulfilment of over 1,100 election pledges in Austria between 1999 and 2008. We draw on coalition theory and related concepts to assess how the preferences of the actors involved and the institutions they employ to police the coalition bargain shape the policy output produced by coalition governments. The results of the multivariate analysis show that institutional constraints are much more powerful predictors of coalition policy than the variables that capture the actors’ preferences.

## Theory and Hypotheses

We put forward two sets of expectations about policy-making in coalition governments. The first refers to the preferences held by the actors involved, whereas the second captures institutional constraints on policy-making in multiparty governments. From a theoretical perspective, both sets of factors are crucial determinants of successful delegation from the party as principal to the coalition cabinet and individual ministers as agents. Yet while the first relates to the causes of delegation problems in coalitions, the second pertains to potential remedies to these problems.

Variation in policy preferences is the root cause of agency loss in multiparty governments (Lupia and Strøm 2008; Müller and Meyer 2010a, 2010b). Whenever parties with different views of the ideal state of the world join together to form a government, there will be areas of substantial policy disagreement. The extent to which party preferences diverge across policy areas is therefore likely to impact on the probability that an election promise will be acted upon. In such situations it is necessary to find common ground. Yet parties may also be more willing to compromise on some issues than on others, depending on which policies are more or less important to them.

The preference divergence between parties translates into (potential) agency loss due to the need to delegate policy implementation from the cabinet collective to individual ministers whose policy positions may diverge substantially from the coalition average (Andeweg 2000; Müller 2000). In anticipation of such delegation problems, parties in coalitions may resort to ex ante and ex post control mechanisms that seek to keep ministerial drift to a minimum (Strøm *et al*. 2010). Ex ante, parties may commit to a common political agenda by setting up and publicising coalition agreements. Even though these documents cannot be legally enforced, empirical studies have highlighted their political importance (Moury 2011a; Timmermans 2003, 2006; Timmermans and Moury 2006). Ex post mechanisms, on the other hand, are installed to prevent future policy actions that deviate from the coalition bargain, the most common example being the assignment of watchdog junior ministers to departments under the control of the coalition partner.

### Preference-Related Determinants

As mentioned above, we start from the premise that policy disagreement is a major factor in coalition politics. The formation of a coalition is conditioned by the preferences of the actors involved, as policy-oriented models of government formation have long argued (Axelrod 1970; de Swaan 1973; Martin and Stevenson 2001; Schofield 1993, 1995; Sened 1996). Also, the lifetime of a government is significantly influenced by its internal preference heterogeneity (Diermeier and Stevenson 1999; Warwick 1992). Furthermore, it has been shown that policy disagreement between parties may hinder or slow down the passage of legislation and the enactment of a political programme (Boranbay *et al.* 2012; Martin and Vanberg 2004, 2005). We test this proposition on two different levels.

First, we have every reason to believe that, whenever all coalition partners have committed themselves to deliver on a specific pledge in their manifestos, we can expect its implementation to face fewer obstacles than in the absence of such cross-party agreement. This notion is also supported by the party mandate literature, which finds that pledges are more likely to be acted upon if there is consensus between the coalition parties (Kostadinova 2013; Thomson 2001).

Since previous studies have already shown that consensual as well as conflicting pledges between governmental parties are an empirically rare phenomenon (Costello and Thomson 2008: 253; Mansergh and Thomson 2007: 315; Royed 1996: 66), we also include a more general measure of policy disagreement. We conjecture that pledge fulfilment should be higher in those areas where the policy distance between the coalition parties is small. A socialist and a liberal party, for instance, may find it easy to agree on the introduction of same-sex marriage but at the same time struggle to implement a coherent economic policy. The two hypotheses referring to policy (dis)agreement therefore read:

**Table:** 

H1a.	A pledge is more likely to be fulfilled if it is supported by all coalition parties.
H1b.	A pledge is more likely to be fulfilled the smaller the distance between the coalition parties on the respective policy dimension.

Political parties differ not only in terms of the positions they take on specific issues, but also in the importance they ascribe to certain policies (Baumgartner *et al*. 2006; Green-Pedersen 2007). In fact, a whole line of research has been developed around the idea that parties compete not by taking diverging positions in the policy space but by emphasising different policy areas (Budge 2001; Budge and Farlie 1983a, 1983b). Our next hypothesis is thus a very simple transfer of this saliency logic to the level of policy pledges: the more important a specific policy proposal to a party, the more likely it is to be implemented. The rationale behind this argument lies in the asymmetric distribution of costs between the parties involved in a coalition government. Putting great emphasis on a specific pledge drives up the (electoral) costs of failing to implement it for the pledge-making party, whereas the other actors’ calculus remains unaffected. Since we expect parties to stress those policies where they are perceived as being especially competent or credible, we can safely assume that it is of particular importance for a party to deliver on those core issues when entering government. We thus conjecture:

**Table:** 

H2.	The more important a pledge is to the pledge-making party the more likely it is to be fulfilled.

A number of studies have also examined the role of opposition parties in forming government policy. Warwick (2001: 1228), for instance, found that the government’s policy position (as stated in government declarations) is significantly influenced by the weighted policy position of all parliamentary parties. In a similar vein, the party mandate literature has produced evidence suggesting that ‘pledges made by government parties are also more likely to be fulfilled when they are in consensus with pledges made by opposition parties’ (Costello and Thomson 2008: 254; see also Kostadinova 2013: 11). The underlying rationale here is that majority support in parliament increases the bargaining power of the pledge-making party vis-à-vis its coalition partners. In addition, some policies, such as constitutional changes, may even require qualified majorities and thus the support of opposition parties. We therefore conjecture:

**Table:** 

H3.	A pledge is more likely to be fulfilled if it has majority support in parliament.

Note that pledges covered by H1a would be a perfect subset of those covered by H3, since all cabinets under study command a majority in parliament. To disentangle the effects, consensual proposals between the coalition parties are excluded in the empirical test of H3.

### Institutional Determinants

Policy-making in coalition governments is not only determined by the preferences of the actors involved. We also consider a number of institutional factors that constrain politicians in their quest to enact their preferred policies.

First, we examine the effect of a control mechanism that parties in coalition bargaining use to bind the prospective government to a specific course of action. Written coalition agreements have become almost ubiquitous in Western European democracies (Müller and Strøm 2008; Strøm and Müller 1999). They provide the public with a comprehensive account of the newly established government’s policy plans and enhance the mutual accountability of the cabinet parties. Since the degree of potential agency loss varies systematically across cabinets, coalition agreements can be explained by structural and preference-related government characteristics as well as the institutional environment (Falcó-Gimeno 2012; Müller and Strøm 2008; Schermann and Ennser-Jedenastik 2012). Furthermore, it has been found that coalition agreements severely constrain governments in their actions (Timmermans 2003, 2006) and serve as a tool to keep ministers in line with the policies agreed upon in the coalition bargain (Moury 2009, 2011a). We therefore assume in H4 that election promises are more likely to be acted upon if they are written down in the coalition agreement.

**Table:** 

H4.	A pledge is more likely to be fulfilled if it is included in the coalition agreement.

In addition to striking a policy bargain, parties in coalitions have to agree on the allocation of ministerial portfolios. Since jurisdiction over a portfolio comes with considerable agenda-setting and veto powers, this can be regarded as one of the most powerful instruments to influence the enactment (or prevention) of a specific policy (Strøm *et al*. 2010: 521). Even so, party leaders are typically granted the freedom to appoint whomever they wish to the cabinet (Müller and Strøm 2000: 574).

Taking the concept of ministerial autonomy to the extreme, Laver and Shepsle (1990; 1996) theorise that cabinet ministers are policy dictators within their jurisdictions, and will therefore implement their party’s ideal policy in the policy area under their control. Critics of this approach argue that cabinets are collective actors that struggle to compromise on a common policy agenda (Dunleavy and Bastow 2001). Nevertheless, it can safely be argued that there is huge potential for agency loss in the delegation of policy from the government as a whole to individual ministers (Andeweg 1993, 1997, 2000). This is because preferences of individual ministers and the cabinet as a collective actor potentially diverge. The allocation of portfolios is therefore one of the main ex ante mechanisms to ensure successful delegation in parliamentary democracies and has been shown to influence the fulfilment of election pledges (Thomson 2001: 191). Ministers can thus be assumed to be much less likely to shirk when tasked with implementing their own party’s policy proposals as opposed to promises made by their coalition partner. This logic is captured in our fifth hypothesis:

**Table:** 

H5.	A pledge is more likely to be fulfilled if the pledge-making party controls the corresponding portfolio.

Of course, parties may anticipate the potential agency loss from delegating to cabinet ministers and employ ex post control mechanisms that keep ministers from deviating too far from the agreed coalition policy. One such tool is the appointment of watchdog junior ministers who are tasked with scrutinising the work of senior ministers and thus ensuring compliance.

Several studies have thoroughly demonstrated the strategic use of such appointments to ‘shadow’ ministers in departments that are of special importance or controlled by parties that are removed from the coalition’s ideal point (Falcó-Gimeno 2012; Lipsmeyer and Pierce 2011; Thies 2001). Furthermore, Ennser-Jedenastik (2013) has shown that watchdog junior ministers do shrink the autonomy of their senior ministers. We can therefore expect that the effect of ministerial discretion (H5) is muted in the presence of a watchdog junior minister. Hypothesis 6 therefore reads:

**Table:** 

H6.	The presence of a watchdog junior minister weakens the effect of ministerial control on pledge fulfilment.

While the content of coalition agreements and the distribution of senior and junior ministerial offices are subject to inter-party negotiations, there is one major institutional constraint that all incoming governments have to accept as their starting point: the policy status quo. At inception each cabinet inherits a myriad of statutes and regulations that are already in place – the ‘dead weight of past policy’ (Warwick 2001: 1217).

A comprehensive governing programme requires decisions about whether to keep or alter the status quo in a multitude of policy areas. Yet altering policy is only possible with the consent of all veto players (Tsebelis 1995, 2002). In the absence of agreement among veto players, the status quo prevails. In coalition governments with no surplus members (such as those examined below), each party is a veto player with the power to block policy changes. A party promising to uphold current policy is therefore in a much better bargaining position than a party seeking changes to the status quo. We therefore argue that the balance of power in coalition governments is tilted toward the parties promising to uphold current policy.^1^

Previous studies of pledge fulfilment have found consistent support for the persistence of the status quo (Costello and Thomson 2008: 250; Kostadinova 2013: 11; Mansergh and Thomson 2007: 319; Royed 1996; Thomson *et al.* 2012: 22). Our seventh hypothesis therefore reads:

**Table:** 

H7.	A pledge is more likely to be fulfilled if it represents the status quo.

The hypotheses put forward above provide the analytical guidelines for our analysis. While some of these assumptions have undergone empirical testing in earlier studies of pledge fulfilment, we present – to the best of our knowledge – the first investigation of pledge saliency (H2), majority support (H3), and the effect of watchdog junior ministers (H6). After discussing the case selection, the next section outlines our mode of operationalisation for the seven hypotheses.

## Data and Methods

The empirical focus of this study is on the fulfilment of pledges in Austria between 2000 and 2008, thus covering three legislative periods (2000–02, 2003–06, and 2007–08) following the elections in 1999, 2002, and 2006. Austria combines cohesive and well-organised parties with a long-standing tradition of two-party coalition governments. Also, the three periods offer some variation in the composition of governments. The Schüssel I cabinet (2000–02) between the conservative Austrian People’s Party (ÖVP) and the right-wing populist Freedom Party (FPÖ) was succeeded by a cabinet of the same partisan make-up (Schüssel II) but the balance of power had shifted dramatically in favour of the ÖVP after its landslide victory at the 2002 snap election. The 2006 general election brought back the grand coalition between the Social Democratic Party (SPÖ) and the ÖVP (Gusenbauer cabinet) that had ruled Austria for much of the post-war era.

The Austrian case, while far from being an outlier among West European democracies, encompasses some interesting characteristics. It combines low partisan turnover in government with relatively high levels of ministerial autonomy, comprehensive coalition agreements, and strong coalition discipline. With respect to some hypotheses (most notably H4, H5, and H7), Austria should therefore be considered a ‘likely case’.

The years from 1999 to 2008 have been selected as a time frame because the election manifestos produced by government parties in those years are, on average, the longest such documents ever produced in Austria (Dolezal *et al*. 2012a), thus providing rich empirical material for the analysis. They can therefore be claimed to provide a comprehensive account of each party’s political programme (Jenny 2006).

To systematically extract data from these texts, we draw on the manifesto analysis scheme developed within the Austrian National Election Study (AUTNES). The AUTNES manifesto analysis (Dolezal *et al*. 2012b, 2013) splits natural sentences into statements based on Noam Chomsky’s phrase-structure model (1957). These statements represent the smallest meaningful building blocks of a sentence. The unitising procedure is thus driven by rules based exclusively on grammar and syntax, meaning that the content of a sentence does not influence the number of statements derived from it. For example, the fictitious sentence ‘We will abolish gift and inheritance taxes’ contains two promises connected by the word ‘and’. This would result in the two separate statements ‘we will abolish the gift tax’ and ‘we will abolish the inheritance tax’. The sentence ‘We guarantee fair, appropriate pensions’ would also result into two separate statements: ‘we guarantee fair pensions’ and ‘we guarantee appropriate pensions’. On average, the researchers extracted 2.4 statements from each sentence (Dolezal *et al.* 2013: 9). The major goal is to provide a most detailed account of the policies put forward in a manifesto.

We apply the widely used definition of Terry Royed (1996: 79), who understands a pledge as a ‘commitment to carry out some action or produce some outcome, where an objective estimation can be made as to whether or not the action was indeed taken or the outcome produced’. Restricting ourselves to this definition of a pledge is a necessary task in order to guarantee the testability of pledge fulfilment. Additionally, this definition is in line with most of the relevant studies in this area (e.g. Artés 2013; Artés and Bustos 2008; Costello and Thomson 2008; Kostadinova 2013; Mansergh and Thomson 2007; Moury 2009; Naurin 2011; Thomson 2001), thus ensuring compatibility across research designs.

The first step of our own data collection consists in identifying pledges in the original manifestos, before transferring the coding onto the data set of AUTNES statements. Based on Royed (1996), we code each statement into one of the following three categories: ‘no pledge’, ‘judgmental pledge’, and ‘definitive pledge’. Descriptions of the status quo, self-praise, or criticism of the political opponent fall into the first category. The middle category includes all statements where parties do indeed make promises to their electorate, but verification would require value judgements to be made (e.g. fair, appropriate pensions). As for the last category, statements are coded as definitive pledges only if the wording allows for an objective assessment as to whether the proposal was in fact implemented or the promised outcome was produced (e.g. abolition of gift and inheritance taxes). The results of this coding process for the government party manifestos can be seen in Table 1.

**Table:** Table 1. Pledges in election manifestos (%)

	1999		2002		2006	
	ÖVP	FPÖ	ÖVP	FPÖ	SPÖ	ÖVP
Statements containing no pledge	59.2	76.2	46.7	66.5	35.7	48.1
Statements containing judgemental pledge	32.7	11.7	41.8	23.5	49.5	41.3
Statements containing definitive pledge	8.1	12.1	11.5	10.1	14.8	10.5

*Note*: Our unit of observation is a statement based on the AUTNES coding procedure. AUTNES splits sentences into shorter statements according to grammatical rules (see discussion in the text).

The figures indicate that, on average, 11 per cent of an election manifesto is reserved for testable promises to their electorate. As for the categories ‘no pledge’ and ‘judgemental pledge’, there is some variation between the six manifestos, yet overall the share of judgemental pledges is around one-third, and just over half of the statements in the average manifesto contain no pledges. Taking into account that a sizeable share of the pledges are made several times by one or even both government parties, this yields a dataset with 1,143 *different* pledges made by the later coalition parties in the run-up to the three elections in 1999, 2002, and 2006.

The authors and two trained graduate students are responsible for the coding of the manifestos. Table 2 presents measures of inter-coder reliability for all six manifestos. The first row reports Krippendorff’s alphas measured on the basis of natural sentences. Applying a commonly used benchmark of α 
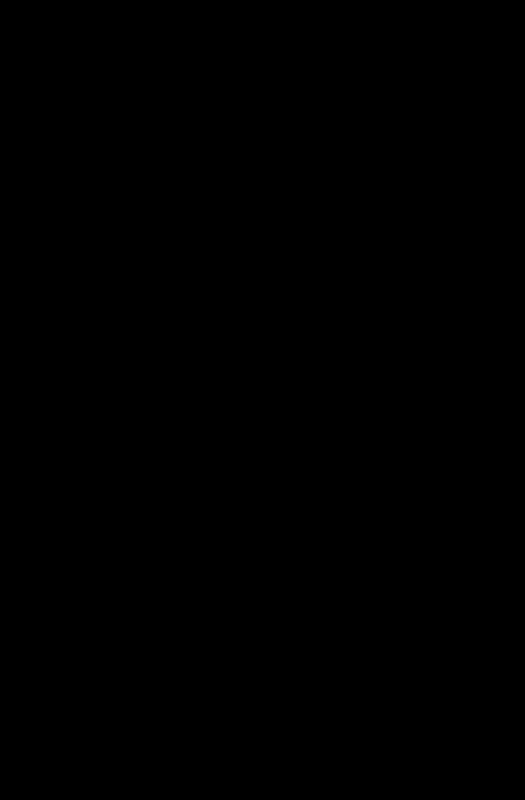
 0.8, one can see that, with the exception of one close outlier (ÖVP 2006), all of the analysed documents satisfy this requirement. The second row presents the percentage agreements regarding the number of definitive pledges identified in the manifestos. The figures are in line with previous studies applying a similar data generating process (Costello and Thomson 2008: 255; Royed 1996: 79; Thomson 2001: 194).

**Table:** Table 2. Inter-coder reliability

	1999		2002		2006	
	ÖVP	FPÖ	ÖVP	FPÖ	SPÖ	ÖVP
Krippendorff’s α (ordinal scale)	0.84	0.95	0.88	0.85	0.85	0.77
Percentage agreement	89.1	95.2	91.0	87.6	90.0	86.1

In order to generate our dependent variable, we then check whether each pledge was not, partly, or fully fulfilled at the end of the legislative period. The coding of pledge fulfilment relies on official statistics (taken mostly from Statistics Austria, Eurostat, or government reports and websites), the legislative database of the Federal Chancellery (www.ris.bka.gv.at) and newspaper accounts found in media archives. If a party was not able to keep its promise, we allocated the pledge to the category ‘not fulfilled’. We coded all pledges that fell short of the promised action or outcome in the category ‘partly fulfilled’ (e.g. a tax cut of 5 per cent when 10 per cent was promised). To be allocated to the ‘fulfilled’ category a measure has to be enacted or the promised outcome produced.

For those pledges whose fulfilment was coded based on annual time series data (e.g. unemployment, inflation, crime, net migration), we established as a baseline the first year of the respective legislative period, to which we compare the average of the following years including the first year of the next legislative period. A promise to lower unemployment in a 1999 manifesto would thus be examined by comparing the unemployment rate in 2000 against the average unemployment between 2001 and 2003.

The following examples should help to make this coding process more transparent. In 2002, the ÖVP promised full tax deductibility of donations for humanitarian and development aid. After a short internet search, we were able to identify several development NGOs that referred to tax deductibility for donations. Also, the Ministry of Finance published on its website a list of tax-advantaged expenditures including reference numbers to the corresponding acts. This identification led us to the legal text in the legislative database of the Federal Chancellery. Since the reform did not enter into force before 2009, the pledge was assigned to the category ‘not fulfilled’.

In their 2006 election manifesto, the Social Democrats committed to lowering the voting age from 18 to 16 years. We started with a general search on the internet and found several online newspaper articles discussing a reform in that matter. Once we knew the official title of the reform, it was easy to pinpoint the corresponding amendment on the parliament’s website. In the last step, we assigned the category ‘fulfilled’ to the pledge.

Table 3 reports the distribution of the pledge fulfilment variable. The multivariate analysis in the next section is based on a dichotomised version of this variable. We combined the two categories ‘partly fulfilled’ and ‘fulfilled’ into one category, ‘at least partly fulfilled’.^2^

**Table:** Table 3. Pledge fulfilment by legislative period (%)

	1999–2002	2003–06	2007–08	Total (*N* = 1,143)
	ÖVP–FPÖ (*N* = 408)	ÖVP–FPÖ (*N* = 460)	SPÖ–ÖVP (*N* = 275)	
Not fulfilled	62.0	43.5	52.0	52.1
Partly fulfilled	3.7	6.1	5.1	5.0
Fulfilled	34.3	50.4	42.9	42.9

*Note*: Due to a lack of data the fulfilment of 96 pledges could not be examined. Typically, such problems arise when no official (or otherwise useable) statistical data are available. A good example is the pledge to decrease the average duration of civil lawsuits, for which no estimates exist prior to 2008.

The figures in Table 3 indicate that the coalition parties managed to at least partly fulfil about 48 per cent of their election pledges. This result is in line with previous studies relying on similar research designs. Analysing election pledges in Ireland, Mansergh and Thomson (2007: 318) reported 50 per cent of at least partially redeemed campaign promises. In the Netherlands, governing parties kept 61 per cent of their promises made during the electoral campaign (Thomson 2001: 191). Unsurprisingly, fulfilment rates in coalition systems are low compared to single party governments. Royed (1996: 61f.) reported 60 per cent of at least partially fulfilled pledges in the United States and 85 per cent in Great Britain, respectively.

The descriptive statistics for the variables are reported in Table 4. The consensus variable indicates whether a pledge was endorsed by both coalition parties in their manifestos. The policy distance variable is generated from the AUTNES manifesto analysis. The AUTNES scheme includes positional measures of party policy on 13 broad policy dimensions (taxes and services, regulation, labour vs. capital, security, social values, multiculturalism, education, environment, urban–rural, Europe, foreign policy, defence, and constitutional issues relating to the diffusion vs. concentration of power within the state). Ranging from –1 to +1, each dimension captures the core policy conflict in the respective area (left- vs. right-wing economic policy, liberal vs. conservative social values, pro- vs. anti-immigration). Each statement in a manifesto is assigned to either pole of the scale (e.g. –1: Eurosceptic vs. +1: pro-European) of a specific dimension (e.g. Europe). The party’s policy position is the mean value across all statements on a dimension and the policy distance represents the absolute difference between the policy positions of the government parties. We use a log-transformed version of this variable to meet the normality assumption.

**Table:** Table 4. Descriptive statistics

Variable	*N*	Mean	SD	Minimum	Maximum
Pledge fulfilment (0 = no, 1 = partly/fully)	1,143	0.479	0.500	0	1
					
Consensus	1,143	0.082	0.275	0	1
Policy distance (logged)	1,100	–3.151	1.529	–7.711	0.430
Pledge saliency (mentioned twice times)	1,143	0.231	0.422	0	1
Pledge saliency (mentioned three times)	1,143	0.084	0.277	0	1
Pledge saliency (mentioned four-plus times)	1,143	0.085	0.279	0	1
Parliamentary majority	1,143	0.093	0.290	0	1
					
Coalition agreement	1,143	0.486	0.500	0	1
Minister, no watchdog junior minister	1,143	0.464	0.499	0	1
Minister plus watchdog junior minister	1,143	0.107	0.309	0	1
Status quo	1,143	0.184	0.387	0	1
					
Period: 2003–06	1,143	0.402	0.491	0	1
Period: 2007–08	1,143	0.241	0.428	0	1
Party: FPÖ	1,143	0.117	0.322	0	1
Party: SPÖ	1,143	0.351	0.477	0	1

*Note*: Since no policy scale exists in the AUTNES scheme for pledges referring to infrastructure, 43 observations are missing for the policy distance variable.

Contrary to previous studies, our measurement of saliency is conducted at the level of single pledges. This is because the length devoted to a policy area in an election programme does not necessarily correspond to the emphasis parties put on a specific pledge within that area. To capture the importance of a single pledge as closely as possible, we therefore counted the number of times it appeared in government parties’ election programmes. Due to the strong right-skewness of this variable we use dichotomous indicators for pledges mentioned two, three, and four or more times (thus the reference category is pledges mentioned once).

Parliamentary support is captured by a dichotomous variable that indicates whether a pledge is supported by a majority in parliament. This information was generated from the coding of opposition party manifestos. Note that all consensual pledges (i.e. agreement between government parties) are coded zero on this variable.

Pledge adoption in the coalition agreement is coded from the coalition agreements that were issued publicly by the three governments (see Schermann and Ennser-Jedenastik 2012).

The hypothesis relating to ministerial control is operationalised by a dummy variable indicating whether the party that made the pledge controls the respective portfolio (e.g. the finance ministry for all pledges referring to taxes). A similar variable is generated for the watchdog junior minister hypothesis. It takes on the value 1 whenever a party holds the responsible minister and this minister is shadowed by a junior minister from the other coalition party. Note that these two indicators are coded to be mutually exclusive.

The status quo variable contains information as to whether a pledge represents the current state of policy and therefore no action whatsoever by the government would be necessary for its fulfilment (e.g. the SPÖ’s pledge in 2006 to leave the corporate income tax unchanged). As control variables we include dummies for parties and legislative periods.

## Analysis

The multivariate analysis of the determinants of pledge fulfilment is presented in Table 5. We inspect the effects of the two sets of expectations about policy-making in coalition governments both separately and combined. The figures indicate clearly that institutional determinants are more powerful predictors than preference-related ones.

The full model (A) predicts more than two-thirds of all cases correctly. Taking the modal category of the dependent variable (52.1 per cent) as reference, this means that the covariates yield not only statistically significant results but also substantively important ones. Likewise, the pseudo R-square (McFadden’s) of around 0.15 in the full model (A) suggests that the independent variables have substantial explanatory power. We also present a full model (B) without the coalition agreement variable, since there are plausible theoretical arguments that this variable is partly endogenous to the process of policy-making. While the bulk of the literature views coalition agreements as representations of the policy bargaining outcome and thus as mechanisms of constraint on the governing parties (Müller and Strøm 2008; Strøm and Müller 1999), it is conceivable that politicians will draft the coalition agreement in anticipation of the most likely policy outcomes over the legislative period. In this case, the optimal estimation strategy would be a simultaneous equation model. However, the problem with this approach is that the most useful covariates to create the required instrumental variable (IV) are, of course, the other independent variables (such as consensus, saliency, status quo, or parliamentary support) which would then disqualify as predictors of pledge fulfilment and thus make testing our main hypotheses unfeasible. The full model (B) in Table 5 reveals that the exclusion of the variable capturing the coalition bargaining outcome does not change our main findings (while decreasing the pseudo R^2^ value).

**Table:** Table 5. Binary logistic regressions: determinants of pledge fulfilment

	Preferences	Institutions	Full model (A)	Full model (B)
Consensus	1.889^∗^		1.141	1.377
	(2.35)		(0.44)	(1.08)
Policy distance (log)	0.848+		0.946	0.911
	(–1.82)		(–0.57)	(–0.97)
Pledge saliency (mentioned twice)	0.999		0.929	0.952
	(–0.01)		(–0.43)	(–0.29)
Pledge saliency (mentioned three times)	1.043		1.242	1.245
	(0.18)		(0.84)	(0.86)
Pledge saliency (mentioned four-plus	1.131		1.139	1.297
times)	(0.48)		(0.48)	(0.96)
Majority (excluding consensual pledges)	1.520+		1.263	1.432
	(1.94)		(0.99)	(1.56)
				
Coalition agreement		2.054^∗∗∗^	1.973^∗∗∗^	
		(5.40)	(4.84)	
Minister, no watchdog		1.624^∗∗∗^	1.612^∗∗^	1.658^∗∗∗^
		(3.36)	(3.13)	(3.35)
Minister plus watchdog		1.488+	1.355	1.440
		(1.76)	(1.27)	(1.54)
Status quo		8.589^∗∗∗^	8.571^∗∗∗^	8.213^∗∗∗^
		(10.39)	(10.14)	(10.04)
				
Party: SPÖ	0.501^∗∗^	0.621+	0.632	0.576^∗^
	(–2.63)	(–1.79)	(–1.62)	(–1.97)
Party: FPÖ	0.486^∗∗∗^	0.655^∗∗^	0.659^∗^	0.633^∗∗^
	(–4.69)	(–2.71)	(–2.44)	(–2.71)
Legislative period: 2003–06	1.430	2.389^∗∗∗^	2.044^∗^	1.815^∗^
	(1.33)	(5.54)	(2.43)	(2.05)
Legislative period: 2007–08	0.896	1.612^∗^	1.305	1.237
	(–0.30)	(2.11)	(0.67)	(0.54)

Observations	1,100	1,143	1,100	1,100
Cases correctly predicted (%)	59.18	67.72	67.82	66.64
McFadden’s *R*^2^	0.045	0.152	0.152	0.136

*Note*: Figures are odds ratios; t-statistics in parentheses; + *p* < 0.1, ^∗^*p* < 0.05, ^∗∗^*p* < 0.01, ^∗∗∗^*p* < 0.001.

Based only on the results of the first model, pledges are more likely to be translated into policy if either the coalition partners agree on the pledge (H1a) or if it enjoys parliamentary support that cuts across the government–opposition divide (H3). An increase of the policy distance between government parties on the other hand, lowers the chances of fulfilment (H1b). Also, the effects of the pledge saliency variables point in the hypothesised direction (H2). The odds ratios above one refer to increased chances of pledge fulfilment, although none of the indicators reach statistical significance.

Turning to the institutional predictors in the second model, there is strong support for the importance of enshrining policy pledges in the coalition agreement (H4). While these documents are not legally binding, they do seem to have a significant political impact on the policy output that is produced by governments. If a pledge makes it from the election manifesto into the coalition agreement, the odds of it being implemented in the following period increase by a factor of two.

The tests of H5 and H6 yield quite interesting results, too. Ministerial control increases the odds of pledge fulfilment by over 60 per cent according to the odds ratios in models 2 and 3. However, while this effect is strong and statistically significant for unconstrained ministers, it is weaker and insignificant (in the full model) for ministers shadowed by a watchdog junior minister. This result is an important qualification of research that has theoretically argued for or empirically demonstrated the importance of individual ministers in influencing policy (Laver and Shepsle 1990, 1996; Thomson 2001). Our data suggest that the appointment of watchdog junior ministers is a real constraint on the effectiveness of ministers to deliver on the promises put forward by their party.

Finally, the status quo (H7) variable has a very strong effect. Indeed, an odds ratio of about 8.6 in a binary logistic regression is quite extraordinary. All else being equal, if a party pledges to maintain the status quo, the odds of (at least partial) fulfilment increase by a factor of 8.6. This result is testament to the ‘stickiness’ of policy that is already in place and the huge bargaining power differential between parties advocating to keep up the status quo and those wanting to repeal it. The vast majority of pledges to enshrine the status quo (which cover about 18 per cent of all pledges) is thus fulfilled, and changes to the status quo against the explicit will of one coalition party are extremely rare. One of the few examples is the FPÖ’s pledge in 2002 to maintain a specific gradual retirement scheme (*Gleitpension*) which was abolished by the Schüssel II cabinet in 2004.

To sum up, the first two models in Table 5 suggest that there is some support for the preference-related hypotheses and quite strong support for the explanatory power of the institutional variables. However, once both sets of variables are taken into account simultaneously, all of the preference-related variables become insignificant. The data thus do not corroborate our first four hypotheses once institutional factors are accounted for. The effects of the latter, on the contrary, remain quite robust in the full model. Substantively, this result indicates that party preferences do not translate directly into policy output but need to be incorporated into the institutional make-up of a coalition government in order to have higher chances of producing the desired policy output. While preferences have been shown to influence the mechanisms of coalition governance that parties employ (Bäck *et al*. 2011; Falcó-Gimeno 2012; Lipsmeyer and Pierce 2011; Müller and Strøm 2008; Schermann and Ennser-Jedenastik 2012), the analysis above implies that it is through the use of these governance mechanisms that preferences are translated into government policy. As a caveat, it should be noted that institutional characteristics are typically easier to measure than preferences (although there can also be ambiguity in assigning pledges to ministerial portfolios or determining the exact nature of the status quo in a policy area). Yet we believe that the potential difference in measurement error cannot account for the difference in explanatory power between institutional variables and preferences.

Finally, the control variables deserve some interpretation. Pledges made by the SPÖ and especially the FPÖ are less likely to be acted upon. While it can be argued that especially the FPÖ had to pay the price for its lack of government experience, this result can also be understood as a consequence of the ÖVP’s greater bargaining power. Accounting for negative coalition signals and the parliamentary arithmetic, the ÖVP had more viable coalition options than any other party at all times during the period of observation. Similarly, there is a straightforward explanation why more pledges were fulfilled in the legislative period between 2003 and 2006, as this was the only time when no early election was called.

**Figure 1:**
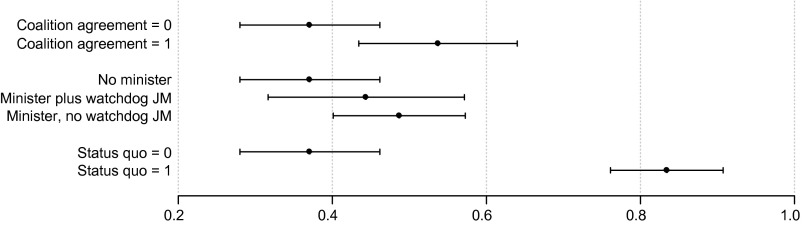
Predicted probabilities of (partial) pledge fulfilment *Note*: Predicted probabilities of (partial) pledge fulfilment with 95 per cent confidence intervals, all other covariates held at their means (continuous variables) or modes (categorical variables); calculations based on full model (A).

In order to demonstrate the effects of the institutional variables more clearly, Figure 1 illustrates the net impact of these predictors. Thus, the impact of individual variables can be assessed more intuitively than from the odds ratios in Table 5. Furthermore, graphical representation also allows for easy comparisons of effect sizes. The predicted probabilities of (partial) pledge fulfilment were calculated based on the full model (A), with all other variables held constant.

The probability of a pledge being (at least partly) implemented rises by 17 per cent (from 37 to 54) as the coalition agreement variable changes from zero to one. Holding the corresponding portfolio without a watchdog junior minister increases the predicted probability of pledge fulfilment from 37 to 49 per cent. However, at 44 per cent the predicted probability is smaller for ‘shadowed’ ministers. Also, the large effect of the status quo is clearly visible in Figure 1. The probability of (partial) fulfilment rises from 37 to 83 per cent as the status quo variable changes from zero to one. This 46 per cent increase underscores that the status quo is an extremely important predictor of government policy.

## Conclusion

Coalition governments pose a challenge to the direct link between a party’s electoral mandate and the policy output produced by a government. Divergent preferences between coalition parties and the intra-cabinet division of labour among ministerial jurisdictions increase the potential for agency loss in the parliamentary chain of delegation (Müller 2000).

In the present study we examine how these problems of delegation play out in the real world and impact on policy-making in multiparty governments. The most important result of our study is that institutional factors dominate party preferences as predictors of coalition policy. Also, we have shown that mechanisms of coalition governance such as coalition agreements or watchdog junior ministers are effective constraints on policy-making.

To be sure, all single-country studies are limited in terms of the extent to which the results generalise to other political systems. While it can safely be argued that Austria is representative for many parliamentary democracies in Western Europe, it may be considered an outlier with respect to some of the factors examined (e.g. regarding ministerial autonomy, see Müller 1994). The generalisability of the result thus hinges upon the extent to which other coalition governments share the characteristics of the Austrian case. This question, however, can only be addressed within a comparative research design that examines patterns of policy-making in coalition governments across a larger number of countries.

## Footnotes

1. In addition to the veto player rationale there are practical and psychological factors that provide politicians with a disincentive to alter the status quo. First, in contrast to the status quo, all proposals to enact policy change suffer from being uncertain to produce the projected outcomes. In the face of such uncertainty, risk-averse politicians may rather opt for the devil they know. Second, since every major policy change is likely to produce winners and losers among their constituents, politicians need to offset support lost among the losers of a decision by pulling enough winners into their camp. However, research on loss aversion and the endowment effect shows that humans dislike losses much more than they value gains of equal size (Kahneman and Tversky 1984; Samuelson and Zeckhauser 1988; Tversky and Kahneman 1991). This predisposition makes changing the status quo electorally risky.

2. Of the 1,143 promises, only 57 are partly fulfilled (see Table 3). A Brant test revealed that our data do not meet the parallel regression assumption of ordinal logistic regression models (*p* = 0.03 in the full model (A)). Therefore, we opt to dichotomise the dependent variable. The results of the analysis are unchanged if these partly fulfilled pledges are allocated to the zero category.

## References

[CIT0001] Andeweg Rudy B., Blondel Jean, Müller-Rommel Ferdinand (1993). A Model of the Cabinet System. The Dimensions of Cabinet Decisionmaking Processes. *Governing Together. The Extent and Limits of Joint Decision-Making in Western European Cabinets*.

[CIT0002] Andeweg Rudy B., Weller Patrick, Bakvis Herman, Rhodes R.A.W. (1997). Collegiality and Collectivity: Cabinets, Cabinet Committees and Cabinet Ministers. *The Hollow Crown. Countervailing Trends in Core Executives*.

[CIT0003] Andeweg Rudy B. (2000). Ministers as Double Agents? The Delegation Process between Cabinets and Ministers. *European Journal of Political Research*.

[CIT0004] Artés Joaquín (2013). Do Spanish Politicians Keep Their Promises?. *Party Politics*.

[CIT0005] Artés Joaquín, Bustos Antonio (2008). Electoral Promises and Minority Governments: An Empirical Study. *European Journal of Political Research*.

[CIT0006] Axelrod Robert (1970). *Conflict of Interest*.

[CIT0007] Bäck Hanna, Debus Marc, Dumont Patrick (2011). Who Gets What in Coalition Governments? Predictors of Portfolio Allocation in Parliamentary Democracies. *European Journal of Political Research*.

[CIT0008] Baumgartner Frank R., Green-Pedersen Christoffer, Jones Bryan D. (2006). Comparative Studies of Policy Agendas. *Journal of European Public Policy*.

[CIT0070] Boranbay, Serra, Thomas E. König, and Sven-Oliver Proksch (2012). ‘A Strategic Model of Coalition Governance in Parliamentary Democracies’, paper prepared for the 2012 Annual Meeting of the American Political Science Association, 30 August–2 September, New Orleans.

[CIT0009] Bräuninger Thomas, Debus Marc (2009). Legislative Agenda-Setting in Parliamentary Democracies. *European Journal of Political Research*.

[CIT0010] Browne Eric, Feste Karen Ann (1975). Qualitative Dimensions of Coalition Payoffs: Evidence From European Party Governments, 1945–1970. *American Behavioral Scientist*.

[CIT0011] Browne Eric, Franklin Mark N. (1973). Aspects of Coalition Payoffs in European Parliamentary Democracies. *American Political Science Review*.

[CIT0012] Browne Eric, Frendreis John (1980). Allocating Coalition Payoffs by Conventional Norms: an Assessment of the Evidence for Cabinet Coalition Situation. *American Journal of Political Science*.

[CIT0013] Budge Ian, Budge Ian, Klingemann Hans-Dieter, Volkens Andrea, Bara Judith, Tanenbaum Eric (2001). Theory and Measurement of Party Policy Positions. *Mapping Policy Preferences. Estimates for Parties, Electors, and Governments*.

[CIT0014] Budge Ian, Farlie Dennis, Daalder H., Mair P. (1983a). Party Competition – Selective Emphasis or Direct Confrontation? An Alternative View with Data. *Western European Party Systems Continuity and Change*.

[CIT0015] Budge Ian, Farlie Dennis J. (1983b). *Explaining and Predicting Elections: Issue Effects and Party Strategies in Twenty-Three Democracies*.

[CIT0016] Chomsky Noam (1957). *Syntactic Structures*.

[CIT0017] Costello Rory, Thomson Robert (2008). Election Pledges and their Enactment in Coalition Governments: A Comparative Analysis of Ireland. *Journal of Elections, Public Opinion and Parties*.

[CIT0018] De Swaan Abram (1973). *Coalition Theory and Cabinet Formation: A Study of Formal Theories of Coalition Formation Applied to Nine European Parliaments After 1918*.

[CIT0019] Diermeier Daniel, Merlo Antonio (2000). Government Turnover in Parliamentary Democracies. *Journal of Economic Theory*.

[CIT0020] Diermeier Daniel, Stevenson Randolph T. (1999). Cabinet Survival and Competing Risks. *American Journal of Political Science*.

[CIT0021] Dolezal Martin, Ennser-Jedenastik Laurenz, Müller Wolfgang C., Winkler Anna Katharina (2012a). The Life Cycle of Party Manifestos: The Austrian Case. *West European Politics*.

[CIT0071] Dolezal, Martin, Laurenz Ennser-Jedenastik, Wolfgang C. Müller, and Anna Katharina Winkler (2012b). ‘Analyzing Manifestos in their Electoral Context: A New Approach with Application to Austria, 2002–2008’, paper presented at the XXIInd World Congress of Political Science (IPSA), Universidad Complutense de Madrid, Spain, July.

[CIT0023] Dunleavy Patrick, Bastow Simon (2001). Modelling Coalitions That Cannot Coalesce: A Critique of the Laver–Shepsle Approach. *West European Politics*.

[CIT0026] Green-Pedersen Christoffer (2007). The Growing Importance of Issue Competition: The Changing Nature of Party Competition in Western Europe. *Political Studies*.

[CIT0027] Jenny Marcelo, Dachs Herbert, Gerlich Peter, Gottweis Herbert, Kramer Helmut, Lauber Volkmar, Müller Wolfgang C., Talos Emmerich (2006). Programme: Parteien im politischen Wettbewerbsraum. *Politik in Österreich. Das Handbuch*.

[CIT0072] Kahneman Daniel, Tversky Amos (1984). Choices, Values, and Frames. *American Psychologist*.

[CIT0028] Kostadinova Petia (2013). Democratic Performance in Post-Communist Bulgaria: Election Pledges and Levels of Fulfillment, 1997–2005. *East European Politics*.

[CIT0029] Laver Michael (2003). Government Termination. *Annual Review of Political Science*.

[CIT0030] Laver Michael, Shepsle Kenneth A. (1990). Coalitions and Cabinet Government. *American Political Science Review*.

[CIT0031] Laver Michael, Shepsle Kenneth A. (1996). *Making and Breaking Governments. Cabinets and Legislatures in Parliamentary Democracies*.

[CIT0032] Lipsmeyer Christine S., Pierce Heather Nicole (2011). The Eyes that Bind: Junior Ministers as Oversight Mechanisms in Coalition Governments. *The Journal of Politics*.

[CIT0033] Lupia, Arthur, and Kaare Strøm (2008). ‘Bargaining, Transaction Costs, and Coalition Governance’, in Kaare Strøm, Wolfgang C. Müller, and Torbjörn Bergman (eds.), *Cabinets and Coalition Government. The Democratic Life Cycle in Western Europe*. Oxford: Oxford University Press, 51–83.

[CIT0034] Mansergh Lucy, Thomson Robert (2007). Election Pledges, Party Competition, and Policymaking. *Comparative Politics*.

[CIT0035] Martin Lanny W. (2004). The Government Agenda in Parliamentary Democracies. *American Journal of Political Science*.

[CIT0036] Martin Lanny W., Stevenson Randolph T. (2001). Government Formation in Parliamentary Democracies. *American Journal of Political Science*.

[CIT0037] Martin Lanny W., Vanberg Georg S. (2004). Policing the Bargain: Coalition Government and Parliamentary Scrutiny. *American Journal of Political Science*.

[CIT0038] Martin Lanny W., Vanberg Georg S. (2005). Coalition Policymaking and Legislative Review. *American Political Science Review*.

[CIT0039] Martin Lanny W., Vanberg Georg S. (2011). *Parliaments and Coalitions. The Role of Legislative Institutions in Multiparty Governance*.

[CIT0040] Moury Catherine (2009). Coalition Government and Party Mandate: Explaining Ministerial Room of Manoeuvre vis-à-vis the Coalition Agreement. *Sociologia, Problemas e Práticas*.

[CIT0041] Moury Catherine (2011). Coalition Agreement and Party Mandate: How Coalition Agreements Constrain the Ministers. *Party Politics*.

[CIT0042] Moury Catherine (2011). Italian Coalitions and Electoral Promises: Assessing the Democratic Performance of the Prodi I and Berlusconi II Governments. *Modern Italy*.

[CIT0043] Müller Wolfgang C., Laver Michael, Shepsle Kenneth A. (1994). Models of Government and the Austrian Cabinet. *Cabinet Ministers and Parliamentary Government*.

[CIT0044] Müller Wolfgang C. (2000). Political Parties in Parliamentary Democracies: Making Delegation and Accountability Work. *European Journal of Political Research*.

[CIT0045] Müller Wolfgang C., Meyer Thomas M. (2010). Meeting the Challenges of Representation and Accountability in Multi-party Governments. *West European Politics*.

[CIT0046] Müller Wolfgang C., Meyer Thomas M., König Thomas, Tsebelis George, Debus Marc (2010). Mutual Veto? How Coalitions Work. *Reform Processes and Policy change: Veto Players and Decision-Making in Modern Democracies*.

[CIT0047] Müller Wolfgang C., Strøm Kaare, Müller Wolfgang C., Strøm Kaare (2000). Conclusion. Coalition Governance in Western Europe. *Coalition Governments in Western Europe*.

[CIT0048] Müller Wolfgang C., Strøm Kaare, Strøm Kaare, Müller Wolfgang C., Bergman Torbjörn (2008). Coalition Agreements and Cabinet Governance. *Cabinets and Coalition Bargaining. The Democratic Life Cycle in Western Europe*.

[CIT0049] Naurin Elin (2011). *Election Promises, Party Behaviour and Voter Perceptions*.

[CIT0050] Riker William H. (1962). *The Theory of Political Coalitions*.

[CIT0051] Royed Terry J. (1996). Testing the Mandate Model in Britain and the United States: Evidence from the Reagan and Thatcher Eras. *British Journal of Political Science*.

[CIT0073] Samuelson William, Zeckhauser Richard (1988). Status Quo Bias in Decision Making. *Journal of Risk and Uncertainty*.

[CIT0053] Schofield Norman (1993). Political Competitition and Multiparty Coalition Governments. *European Journal of Political Research*.

[CIT0054] Schofield Norman (1995). Coalition Politics: A Formal Model and Empirical Analysis. *Journal of Theoretical Politics*.

[CIT0055] Sened Itai (1996). A Model of Coalition Formation: Theory and Evidence. *The Journal of Politics*.

[CIT0056] Strøm Kaare, Müller Wolfgang C. (1999). The Keys to Togetherness: Coalition Agreements in Parliamentary Democracies. *Journal of Legislative Studies*.

[CIT0057] Strøm Kaare, Müller Wolfgang C., Smith Daniel Markham (2010). Parliamentary Control of Coalition Governments. *Annual Review of Political Science*.

[CIT0058] Thies Michael F. (2001). Keeping Tabs on Partners: The Logic of Delegation in Coalition Governments. *American Journal of Political Science*.

[CIT0059] Thomson Robert (2001). The Programme to Policy Linkage: The Fulfilment of Election Pledges on Socio-Economic Policy in the Netherlands, 1986–1998. *European Journal of Political Research*.

[CIT0074] Thomson, Robert, Terry Royed, Elin Naurin, Joaquin Artes, Mark J. Ferguson, Petia Kostadinova, and Catherine Moury (2012). ‘The Program-to-Policy Linkage: A Comparative Study of Election Pledges and Government Policies in Ten Countries’, paper prepared for the 2012 Annual Meeting of the American Political Science Association, 30 August–2 September, New Orleans.

[CIT0060] Timmermans Arco (2003). *High Politics in the Low Countries. An Empirical Study of Coalition Agreements in Belgium and The Netherlands*.

[CIT0061] Timmermans Arco (2006). Standing Apart and Sitting Together: Enforcing Coalition Agreements in Multiparty Systems. *European Journal of Political Research*.

[CIT0062] Timmermans Arco, Moury Catherine (2006). Coalition Governance in Belgium and The Netherlands: Rising Government Stability Against All Electoral Odds. *Acta Politica*.

[CIT0063] Tsebelis George (1995). Decision Making in Political Systems: Veto Players in Presidentialism, Parliamentarism, Multicameralism and Multipartyism. *British Journal of Political Science*.

[CIT0064] Tsebelis George (2002). *Veto Players. How Political Institutions Work*.

[CIT1997] Tversky Amos, Kahneman Daniel (1991). Loss Aversion in Riskless Choice: A Reference-Dependent Model. *The Quarterly Journal of Economics*.

[CIT0065] Warwick Paul (1992). Ideological Diversity and Government Survival in Western European Parliamentary Democracies. *Comparative Political Studies*.

[CIT0066] Warwick Paul (1994). *Government Survival in Parliamentary Democracies*.

[CIT0067] Warwick Paul (2001). Coalition Policy in Parliamentary Democracies. Who Gets How Much and Why. *Comparative Political Studies*.

[CIT0068] Warwick Paul, Druckman James (2001). Portfolio Salience and the Proportionality of Payoffs in Coalition Governments. *British Journal of Political Science*.

[CIT0069] Warwick Paul, Druckman James (2006). The Portfolio Allocation Paradox: An Investigation into the Nature of a Very Strong but Puzzling Relationship. *European Journal of Political Research*.

